# Pharmacological Enhancement of Dopamine Neurotransmission Does Not Affect Illusory Pattern Perception

**DOI:** 10.1523/ENEURO.0465-23.2024

**Published:** 2024-07-19

**Authors:** Elke Smith, Simon Michalski, Kilian Knauth, Deniz Tuzsus, Hendrik Theis, Thilo van Eimeren, Jan Peters

**Affiliations:** ^1^Department of Psychology, Biological Psychology, University of Cologne, Cologne 50969, Germany; ^2^Faculty of Medicine and University Hospital Cologne, Department of Nuclear Medicine, University of Cologne, 50937 Cologne, Germany; ^3^ Faculty of Medicine and University Hospital Cologne, Department of Neurology, University of Cologne, 50937 Cologne, Germany

**Keywords:** discrimination sensitivity, dopamine, ʟ-DOPA, pattern perception

## Abstract

Psychotic symptoms and delusional beliefs have been linked to dopamine transmission in both healthy and clinical samples and are assumed to result at least in part from perceiving illusory patterns in noise. However, the existing literature on the role of dopamine in detecting patterns in noise is inconclusive. To address this issue, we assessed the effect of manipulating dopaminergic neurotransmission on illusory pattern perception in healthy individuals (*n *= 48, *n *= 19 female) in a double-blind placebo-controlled within-subjects design (see preregistration at https://osf.io/a4k9j/). We predicted individuals on versus off ʟ-DOPA to be more likely to perceive illusory patterns, specifically objects in images containing only noise. Using a signal detection model, however, we found no credible evidence that ʟ-DOPA compared with placebo increased false alarm rates. Further, ʟ-DOPA did not reliably modulate measures of accuracy, discrimination sensitivity, and response bias. In all cases, Bayesian statistics revealed strong evidence in favor of the null hypothesis. The task design followed previous work on illusory pattern perception and comprised a limited number of items per condition. The results therefore need to be interpreted with caution, as power was limited. Future studies should address illusory pattern perception using more items and take into account potential dose-dependent effects and differential effects in healthy versus clinical samples.

## Significance Statement

Psychosis and delusional belief have been linked to dopamine transmission in healthy and clinical samples and are assumed to partly result from perceiving illusory patterns in noise. However, the findings on the role of dopamine in detecting illusory patterns are inconclusive. To address this, we assessed the effect of enhancing dopamine transmission on illusory pattern perception in healthy individuals. Our hypothesis that enhancing dopamine transmission would increase participants’ tendency to perceive illusory patterns in noise was not confirmed. This null effect suggests that earlier findings may be less robust than previously thought and that the relationship between dopamine and illusory pattern perception may be subject to dose-dependent effects and that there may be differential effects in healthy versus clinical samples.

## Introduction

Detecting relationships between stimuli or events enables individuals to make predictions for the future. Recent theories conceive the brain as a probabilistic inference system, predicting events and causes of sensory input to enable successful interaction with the environment (Friston and Stephan, 2007). Perceiving relationships between unrelated stimuli and patterns in noise may be maladaptive, however, since this prevents the formation of accurate representations. Delusional belief and psychotic symptoms are assumed to result from aberrant changes in dopaminergic signaling. Several studies point toward a link between dopamine transmission and delusional belief in healthy and clinical samples. For instance, manipulating dopamine transmission with haloperidol and ʟ-DOPA in controls changed social attributions of harmful intent (Barnby et al., 2020). Further, the link between dopamine and delusions is supported by the effects of antipsychotics, which alleviate psychotic symptoms by antagonizing D_2_ dopamine receptors (Kaar et al., 2020) and by PET imaging studies showing dysregulated dopamine synthesis in individuals suffering from delusions and schizophrenia (Cheng et al., 2020; for a review, see Rigney et al., 2021).

According to prevailing theories, this link is imparted by dopamine's role in the encoding of reward prediction errors (RPEs) and the assignment of aberrant salience. Within the framework of the prediction error minimization theory, the brain seeks to minimize the discrepancy between predicted and actual input (Clark, 2013). The theory has been widely adopted for describing decision-making in various domains, including reward-based learning (Schultz, 2016), perceptual (Bell et al., 2016), and social inference (de Bruin and Michael, 2021). A large body of research suggests that dopamine modulates striatal coding of prediction errors to enable learning (Pessiglione et al., 2006; Glimcher, 2011; Schlagenhauf et al., 2013; Schultz, 2016; Basanisi et al., 2023). Imaging studies in human schizophrenic participants have reported striatal dysfunction during learning (Schlagenhauf et al., 2014; Katthagen et al., 2020) and aberrant learning from feedback accompanied by altered EEG correlates (Kirschner et al., 2024). In monkeys, midbrain dopamine neurons have been found to code reward size but also uncertainty or confidence during perceptual decision-making (de Lafuente and Romo, 2011; Lak et al., 2017).

The aberrant salience framework of psychosis describes delusions as a dysfunctional computational mechanism at the neural level within a Bayesian predictive coding framework (Kapur, 2003). Representations are formed by weighting prior beliefs against sensory inputs based on their probabilities (Sterzer et al., 2018). Psychosis is assumed to result from low precision of prior beliefs and dysfunctional belief updating, conditioned by changes in dopamine signaling (Heinz et al., 2019). In mice, increasing striatal dopamine levels related to poor precision, i.e., high-confidence false alarms, in an auditory stimulus detection task, regarded as hallucination-like perceptions, (Schmack et al., 2021). A hyperdopaminergic state is thought to further cause aberrant assignment of salience to formed representations. Delusional beliefs reflect an individual's cognitive effort to explain experiences of aberrant salience, while hallucinations reflect the experience of aberrantly salient internal representations (Kapur, 2003).

Conceptually, paranormal belief, conspiratorial thinking, and schizotypy represent nonpathological states on a continuum converging toward delusional belief and psychosis (Kreweras, 1983; Denovan et al., 2018). For instance, conspiratorial beliefs, i.e., beliefs that certain events result from secret plots by powerful actors, correlated positively with paranormal beliefs, paranoid ideation, and schizotypy (Darwin et al., 2011). Interestingly, for both the social and perceptual domain, dopamine has been linked to delusional belief and psychotic symptoms in healthy and clinical samples (Sekine et al., 2001; Howes and Kapur, 2009; Krummenacher et al., 2010; Barnby et al., 2020). For the perceptual domain, studies assessing the ability to discriminate signals and noise in controls and individuals with hallucinations and schizophrenia yielded mixed results (Bentall and Slade, 1985; Ishigaki and Tanno, 1999; Krummenacher et al., 2010). Early studies report a more liberal criterion, i.e., a tendency to identify signals, but no difference in discrimination sensitivity, for individuals with high compared with low predisposition to hallucination in auditory signal detection (Bentall and Slade, 1985), and decreased discrimination sensitivity in patients with schizophrenia and auditory hallucinations compared with controls in a visual continuous performance test (Ishigaki and Tanno, 1999). In contrast, a more recent study reports individuals with paranormal beliefs to favor false alarms over misses and individuals skeptical about paranormal phenomena to show the reverse strategy. Enhancing dopaminergic neurotransmission lowered discrimination sensitivity compared with placebo in skeptics, but not believers (Krummenacher et al., 2010).

Dopamine has been assumed to reduce noise distortion in neuronal signal transmission (Walter and Spitzer, 2003; Vander Weele et al., 2018). However, considering the above findings and psychosis as hyperdopaminergic state characterized by a poor ability to discriminate relevant and irrelevant, and internal and external stimuli (Morris et al., 2013; Chu et al., 2021), this assumption falls short. The inconsistencies might be related to sample characteristics, for instance, to differences in predisposition to delusional thinking or symptomatology, or to the domain under study, such as auditory or visual. Further, most of the studies rely on rather small subgroup samples (Ishigaki and Tanno, 1999; Krummenacher et al., 2010; Barnby et al., 2020).

In view of these inconclusive findings, we aimed at investigating whether enhancing dopamine transmission elicits delusional beliefs already in healthy controls at an “early” perceptual (in contrast to, for instance, the cognitive process of establishing connections between events by means of complex explanations) stage, i.e., in visual perception. To this end, we used a pharmacological approach in healthy controls, increasing dopamine transmission with the dopamine precursor ʟ-DOPA, and studied the effects on illusory visual pattern perception. Modeling discrimination performance with signal detection theory, we predicted participants on versus off ʟ-DOPA to exhibit increases in false alarms, i.e., to perceive more illusory patterns, specifically objects in images containing only noise (see preregistration at https://osf.io/a4k9j/). With regard to response bias, discrimination sensitivity and accuracy, our hypotheses were nondirectional.

## Materials and Methods

### Ethics statement

The study was approved by the local ethics committee of the Faculty of Medicine of the University of Cologne, Germany.

### Sample

A subset of *n *= 49 out of *N *= 76 participants from a larger pharmacological study performed a visual perception task, specifically the snowy pictures task (SPT; Ekstrom and Harman, 1976; Whitson and Galinsky, 2008). One participant was excluded due to side effects (nausea and vomiting) and did not complete the task. The final sample analyzed here included 48 participants, including 19 women, all right-handed, aged 25–40 (*M *= 28.27). The participants were recruited in Cologne, Germany. They were recruited through university bulletins, through mailing lists, and by word-of-mouth recommendation. For practical reasons, only a subset of participants completed the SPT. Therefore, no task-specific a priori power calculation was carried out. A post hoc power analysis (paired samples test with G*Power, version 3.1.9.7; Faul et al., 2009) yielded a power of 0.39 to detect a small effect (*d *= 0.2), power of 0.96 to detect a medium effect (*d *= 0.5), and a power of >0.99 to detect a large effect (*d *= 0.8). All participants had normal or corrected-to-normal vision, German as first language (or profound German language skills), and all women were taking hormonal contraceptives. General exclusion criteria for study participation were strongly impaired vision or strabismus, participation in other studies involving medications, intake of nonprescription and prescription drugs, pregnancy, acute infections, alcohol or drug intoxication or abuse, psychiatric disorders (past or current), neurological disorders, metabolic disorders, internal diseases, chronic pain syndrome, complications of anesthesia, and strong emotional burden or physical stress during the study period. Exclusion criteria considering contraindications regarding the intake of ʟ-DOPA were hypersensitivity to ʟ-DOPA or benserazide, intake of nonselective monoamine oxidase inhibitors, metoclopramide or antihypertensive medication (e.g., reserpine), disorders of the central dopaminergic system, e.g., Lewy body dementia or Parkinson's disease, increased intraocular pressure (glaucoma), and breastfeeding.

### Procedure

The current study was part of a larger pharmacological project assessing dopamine effects on decision-making and learning. Since the research questions, tasks and methods used are fundamentally different, reporting on all projects would go beyond the scope of the current article. Therefore, we focus on the analysis of the visual perception task and will report on the other projects elsewhere. After passing a medical examination by a physician to check for contraindications, the participants were invited to three testing sessions. During the first session, the participants underwent a baseline screening for putative dopamine proxies, specifically working memory capacity, spontaneous eyeblink rate, and impulsivity (Gibbs and D’Esposito, 2005; Dalley et al., 2007; Jongkees and Colzato, 2016). Since the investigation of the influence of putative proxies of dopamine function was only conducted for the effects of ʟ-DOPA on intertemporal choice and reinforcement learning, the data from the baseline screening will be reported elsewhere. After the baseline screening, the participants completed two identical experimental sessions on separate days with an interval of approximately a week between the sessions. Thirty minutes prior to testing, the participants received a nondistinguishable tablet containing either 150 mg ʟ-DOPA, a dopamine precursor, and benserazide, a peripheral decarboxylase inhibitor, or placebo and then completed an intertemporal choice task and a reinforcement learning task (see preregistration at https://osf.io/a4k9j/). Seventy-six participants completed the intertemporal choice and reinforcement task. Approximately 75 min after intake of the tablet, a subset of participants (*n *= 49 out of *N *= 76; see below, Snowy pictures task) additionally completed a visual perception task. The study was realized as double-blind placebo-controlled within-subjects design. Polling the participants showed that they could not guess the correct order of the experimental sessions *χ*^2^ (1, *N *= 48) = 0, *p *= 1.0.

### Snowy pictures task

We used a modified pen-and-paper version of the SPT (Ekstrom and Harman, 1976; Whitson and Galinsky, 2008). The task contains 24 grainy images. Some of the images contain hard-to-detect embedded objects (for instance, a chair and a knife), and some contain only noise (12 images with objects, 12 images with noise). The participants were asked to denote whether or not an object is present in the image, and if so, what object it is. They were instructed to complete the task as fast as possible without sacrificing accuracy. The participants completed two different versions of the task under placebo and ʟ-DOPA, respectively, in counterbalanced order (12 images per session).

### Data analysis

To assess the influence of enhancing dopaminergic transmission on the detection of objects in images, we calculated accuracies, false alarm rates, and the signal detection theory measures *d*-prime and response bias per condition (placebo and ʟ-DOPA). *D*-prime is an index for the ability to disentangle signal from noise, with higher values reflecting greater discriminability, while the response bias reflects the tendency toward responding “yes” or “no.” Negative values indicate a liberal response criterion (response bias toward responding “yes”), while positive values indicate a conservative response criterion (response bias toward responding “no”). *D*-prime and response bias were computed participant- and condition-wise in MATLAB (version R2023a) based on Stanislaw and Todorov (1999). Following the 1/2N rule, we corrected perfect hit and false alarm rates by $-1/(2n_{\mathrm{target}})$ and $+1/(2n_{\mathrm{distractor}})$, respectively (Stanislaw and Todorov, 1999). Significant Shapiro–Wilk tests for accuracy *W *= 0.928, *p *= 0.006; false alarm rate *W *= 0.909, *p *= 0.001; and response bias differences *W *= 0.934, *p *< 0.009 indicated that differences between the matched pairs were not normally distributed. Therefore, we used nonparametric Bayesian Wilcoxon signed-rank tests as implemented in JASP (version 0.17.2.1) to compare the group means of accuracy, false alarm rate, and response bias between conditions. Since *D*-prime was normally distributed (*W *= 0.971; *p *= 0.270), we used a Bayesian paired samples *t* test to compare *d*-prime between conditions. The posterior distributions were obtained using Markov chain Monte Carlo (MCMC) sampling with five chains and 1,000 samples, using a Cauchy distribution with scale = 2 as prior. We report Bayes factors to evaluate evidence in favor of the null hypothesis (nondirectional, *BF*_01_; directional, *BF*_0−_).

We further modeled the decisions with a hierarchical signal detection model (SDT) using Bayesian inference. The model was implemented using the *bhsdtr* package (version 2; Paulewicz and Blaut, 2020) for R (version 4.1.2; R Core Team, 2021). The model implements a hierarchical regression structure on the SDT parameters and accounts for parameter variability due to factors such as participants and items. The hierarchical general linear regression structure for the SDT parameters requires the parameters to be unconstrained. More specifically, to account for the assumption of normally distributed random effects, the model is reparameterized such that the parameters are unconstrained (since the normal distribution is unbounded). *D*-prime (*d*′) is derived from $\delta =\ln(d^{\prime })$, allowing random effects to be modeled by assuming that *δ* is normally distributed. For a full description of the model, the reader is referred to Paulewicz and Blaut (2020). For *d*-prime, we modeled the drug effect as fixed effect and participants and items as random effects. For the threshold (response bias), we modeled the drug effect as fixed effect and participants as random effects:

δ=∼drug+(1|id)+(1|trial),

and

(.) γ=∼drug+(1|id).

For the priors on the random effects correlations, the *bhsdtr* package implements Cholesky decomposition of the correlation matrices, with uniform priors by default. For both *d*-prime and the threshold, we used normal priors for fixed effects (with μ=0.5, σ=1 and μ=0.5, σ=1, respectively) and uniform priors for random effects. To assess a possible drug effect on *d*-prime and the threshold, we calculated the Savage–Dickey density ratio for the posterior difference distributions for placebo versus ʟ-DOPA by dividing the value of the posteriors over the parameters evaluated at θ=0 by the priors. Sampling was performed with Hamiltonian Monte Carlo sampling, using the *Stan* modeling language (Stan Development Team, 2023) via the *rstan* interface (version 2.32.2; Stan Development Team, 2024), with four chains, 1,000 warmup samples, and 2,000 iterations. We determined chain convergence by inspecting the traceplots and accepting values of $\hat{R}\leq 1.01$ (Gelman and Rubin, 1992).

### Code and data accessibility

The data and the code used to analyze the data are freely available online at https://osf.io/m5u6v/ and https://osf.io/m7g3p/, respectively.

## Results

**Figure 1.**
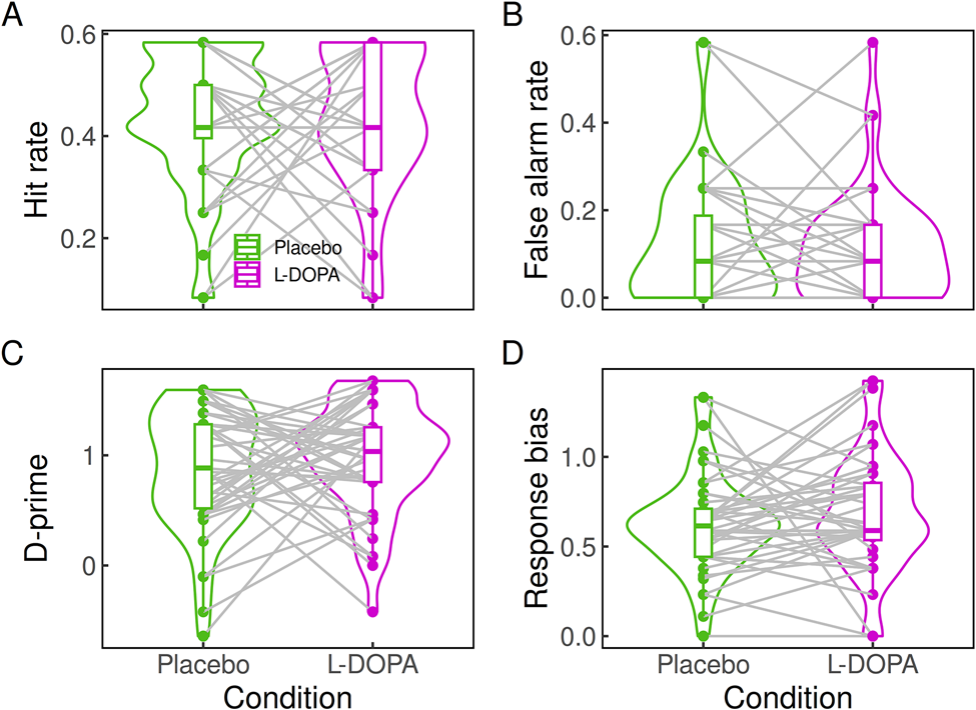
Distribution of accuracy (***A***), false alarm rate (***B***), *d*-prime (***C***), and response bias (***D***) per condition. Horizontal line, median; box, first and third quartiles; lower whisker, lowest value not ∼1.5*IQR (interquartile range) from the first quartile; upper whisker, highest value no further than 1.5*IQR from the third quartile; dots, outliers (data points outside the lower and upper whisker). For the hit rate and false alarm rate (panels ***A*** and ***B***, respectively), there are overlapping data points.

**Figure 2.**
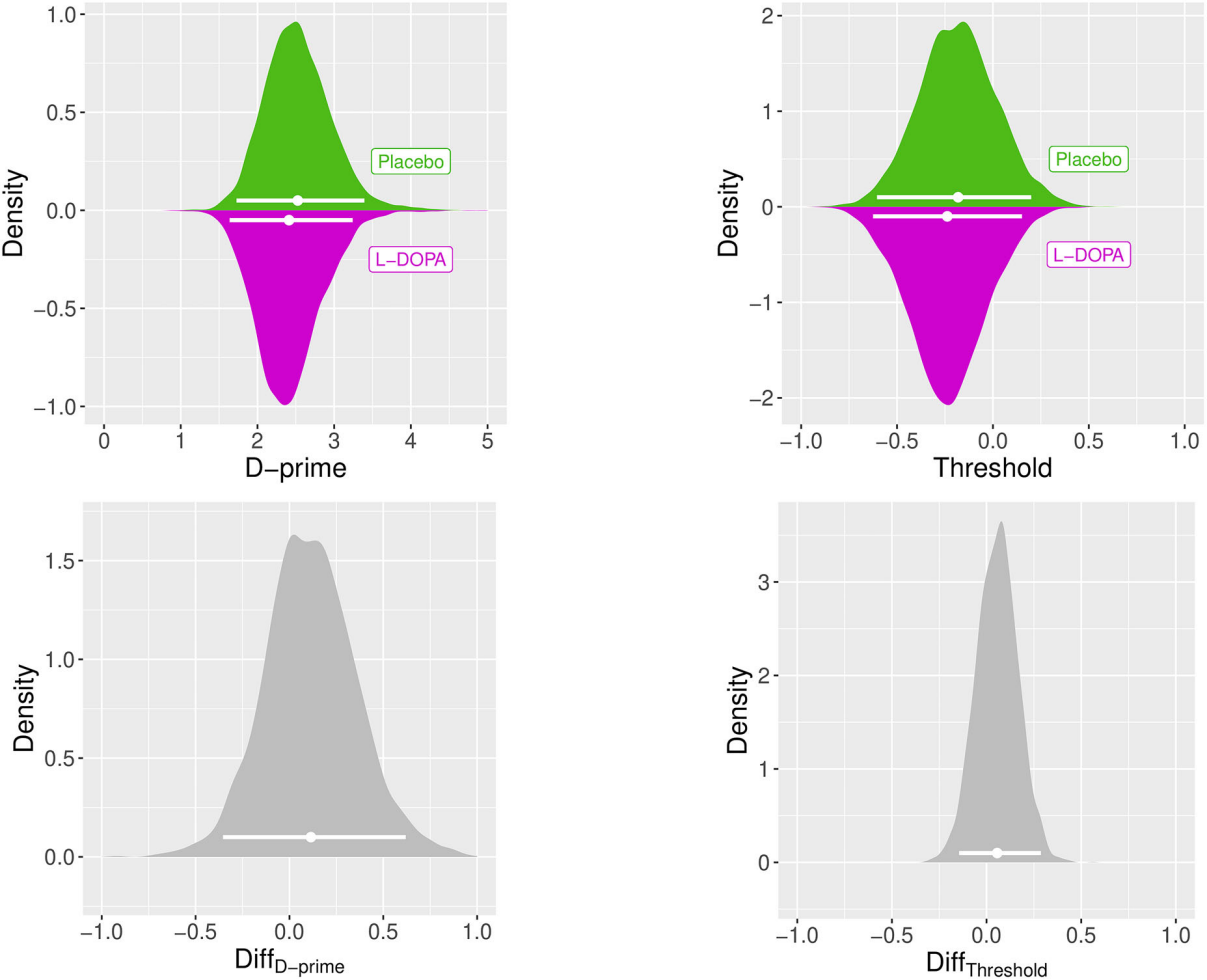
Top row, Group-level posterior distributions for d-prime and threshold (response bias), for the placebo (green, top) and ʟ-DOPA (magenta, bottom) condition. Bottom row, Difference distributions. The horizontal solid lines indicate the 95% highest posterior density intervals.

On average, and across both conditions (placebo and ʟ-DOPA), participants correctly detected objects or correctly rejected noise in 81.77% of all images. The average response bias across both conditions of 0.65 indicates an overall tendency toward responding “no” (i.e., no object identified in an image). Under ʟ-DOPA, participants made false alarms in 11.28% of all images, i.e., identified objects in images containing only noise, compared with 12.15% in the placebo condition (see Table 1 and Fig. 1 for accuracy, hits, false alarms, *d*-prime, and response bias per drug condition). Testing for group differences in accuracy, false alarm rate, response bias, and *d*-prime between the conditions revealed that the null effect of no difference in accuracy under ʟ-DOPA and placebo was 14 times more likely than a difference between the conditions (*BF*_01_ = 14.48). Likewise, a null effect for the false alarm rate between ʟ-DOPA and placebo was 20 times more likely than an increased false alarm rate under ʟ-DOPA versus placebo (*BF*_0−_ = 20.25). Furthermore, Bayesian analyses revealed evidence in favor of the null hypothesis for the response bias (*BF*_01_ = 6.37) and *d*-prime (*BF*_01_ = 11.46). These results were confirmed when analyzing the data using a hierarchical Bayesian implementation of the SDT (Paulewicz and Blaut, 2020) that may be more robust, given the low trial numbers in individual participants. The group-level posterior distributions for *d*-prime and threshold are depicted in Figure 2. Given the priors and the data, null effects for the drug effect on *d*-prime and threshold (response bias) were more likely than the alternative (BF_01_ = 7.29 and BF_01_ = 47.76, respectively).

**Table 1.** Means and standard deviations for task performance per drug condition

	Drug condition			
	Placebo		ʟ-DOPA	
	*M*	SD	*M*	SD
Accuracy	0.81	0.13	0.83	0.12
Hit rate	0.43	0.13	0.44	0.14
False alarm rate	0.12	0.14	0.11	0.12
*D*-prime	0.89	0.54	0.98	0.48
Response bias	0.63	0.27	0.68	0.33

## Discussion

We studied the effects of enhancing dopamine neurotransmission using the dopamine precursor ʟ-DOPA on illusory pattern perception using a visual perception task. Participants completed two versions of the modified SPT (Ekstrom and Harman, 1976; Whitson and Galinsky, 2008) under placebo and ʟ-DOPA, respectively. Applying signal detection theory, our hypothesis that participants on ʟ-DOPA would be more likely to perceive illusory patterns, specifically objects in images containing only noise was not confirmed. In contrast, Bayesian analyses revealed strong evidence in favor of the null hypothesis for false alarm rates. Likewise, for accuracy, discrimination sensitivity, and response bias, Bayesian analyses revealed evidence in favor of the null hypothesis.

Changes in discrimination sensitivity and social attributions relevant to paranoia following dopaminergic modulation have previously been reported in perceptual and social decision-making in healthy samples (Krummenacher et al., 2010; Barnby et al., 2020). The involvement of dopamine in delusional ideation is further substantiated by the efficacy of antipsychotic treatment (Kaar et al., 2020), and a study assessing perceptual discrimination in controls and individuals with hallucinations and schizophrenia reported lower discrimination sensitivity in patients with auditory hallucinations (Ishigaki and Tanno, 1999). The present null effect of ʟ-DOPA on illusory pattern perception in the current study may be for several reasons. First, earlier studies reporting a relationship between dopamine and discrimination sensitivity or paranoid inferences rely on rather low sample sizes [30 participants in within-subjects design in Barnby et al. (2020), 20 participants per belief group in between-subject design in Krummenacher et al. (2010), 11 participants per patient group in Ishigaki and Tanno (1999)]. Low sample sizes increase the variance in effect sizes even under the null, such that previously reported findings may have been false positives. Alternatively, the present null effect may be related to dose-dependent effects or a single dose may have not been sufficient to elicit detectable changes in pattern perception. The effect of pharmacological DA manipulation might also depend on interindividual differences, such as a predisposition to delusional thinking, belief in the paranormal (Krummenacher et al., 2010), magical ideation (Mohr et al., 2006), and predisposition to hallucinations (Bentall and Slade, 1985). For instance, ʟ-DOPA increased semantic priming only in participants with high magical ideation (due to longer response times for unrelated prime-target pairs), and participants with high magical ideation under placebo performed comparable with participants with low magical ideation under ʟ-DOPA (Mohr et al., 2006). It is further conceivable that dopamine is related to delusional beliefs, while manifesting itself only in the pathological state or in individuals scoring high on schizotypy (Mohr and Ettinger, 2014). Lastly, the nature of the task may not be suitable to detect dopamine-related changes in illusory pattern perception, since it covers the visual domain only, and requires no inferences about events or social intentions.

### Limitations

The current study has some limitations. Plasma levels peak 30–60 min after intake of ʟ-DOPA, while plasma half-life is ∼90 min (Hauser, 2009; Keller et al., 2011). Being part of a larger project, the present task was performed following two other behavioural tasks. The task was completed ∼75 min after intake and was typically completed in 5–10 min. Accordingly, the task was completed after plasma levels had peaked, but likely before the plasma half-life was reached. Still, the timing, and therefore the dose during task performance, may have contributed to the null effects of ʟ-DOPA on pattern perception. Dose-dependent effects on perceptual decision-making have been reported in another study, which modulated dopamine transmission via methylphenidate (Beste et al., 2018). Further, we did not assess the participants’ baseline dopamine synthesis capacity and their predisposition to delusional thinking or paranormal belief. The participants in our sample may not have had such a predisposition, and a single dose of ʟ-DOPA may therefore not have been sufficient to induce such effects. Also, since overall accuracy was rather high, and false alarm rates rather low, the task may be susceptible to ceiling effects.

The SPT has been frequently used to study illusory pattern perception. However, the task comprises a comparatively small number of items. In the original publication, using a between-subjects design, Whitson and Galinsky (2008) report differences between experimental conditions (lack of control vs baseline) using the same number of items per condition as in the present study. Using the same task, group differences in task performance between controls and patients with schizophrenia have been reported (Moritz et al., 2014), within-subject differences in terms of higher confidence in errors under ʟ-DOPA compared with placebo (Andreou et al., 2015), and associations between illusory pattern perception in the SPT and conspiracy belief (Hartmann and Müller, 2023). Of note, however, a more recent study (van Elk and Lodder, 2018) found no evidence that loss of control affects illusory pattern perception in the SPT. A strength of the current study is the within-subjects design, yielding higher power compared with between-subjects designs. Still, the low number of items may have limited power so as to detect small to medium effects. Furthermore, we additionally implemented a hierarchical Bayesian SDT model that might be more robust given the low number of trials per participant. This model confirmed the original result, such that null effects were substantially more likely given the data. Hierarchical Bayesian models also come with some limitations. Such models are sensitive to the choice of the prior distribution. Using hierarchical Bayesian modeling, we assume a specific type of distribution for the variability across the group, which may fall short in some cases (McGlothlin and Viele, 2018).

### Conclusion and perspectives

We assessed the effect of enhancing dopaminergic neurotransmission on illusory pattern perception. There was no evidence that ʟ-DOPA, compared with placebo, increases the detection of patterns in noise. Rather, Bayesian analyses provided strong evidence in favor of the null hypothesis. Future studies should control for predisposition to delusional thinking and belief in the paranormal or magical ideation and may assess changes in pattern perception across different domains (e.g., auditory and visual).

## Data Availability

The data that support the findings of this study are openly available at https://osf.io/m5u6v/. The study protocol was preregistered (see https://osf.io/a4k9j/).

## Synthesis

Reviewing Editor: Frederike Beyer, Queen Mary University of London Decisions are customarily a result of the Reviewing Editor and the peer reviewers coming together and discussing their recommendations until a consensus is reached. When revisions are invited, a fact-based synthesis statement explaining their decision and outlining what is needed to prepare a revision will be listed below. The following reviewer(s) agreed to reveal their identity: Stijn Nuiten.

As you can see from their comments below, the reviewers appreciate the efforts that have gone into the revision of this manuscript. However, as evident from our dsicussion on this, we all agree the low trial number seriously limits the conclusions that can be drawn based on the presented results. As such, if further data collection to address this issue is not an option, the article needs to state clearly and early on (i.e. already in the abstract) what the limitations of this study are, and that the results need to be interpreted with caution.

Besides this, further information on the bayesian analyses conducted and some more detail in other areas is required for the article to be acceptable for publication. Please see detailed comments of the reviewers below.

Best wishes,

Frederike Beyer

Reviewer 1 comments

Although the trial counts for each participant remain quite low, the newly added hierarchical Bayesian modelling does add more credence to the null-findings reported in this manuscript. Besides this addition, the manuscript has been sufficiently revised, particularly regarding previous literature and detailing of the methods.

Reviewer 2 comments

The authors argue that levodopa has no effect on the illusory visual pattern perception in the Snowy Pictures Task. This is of potential interest to research into perception, psychosis, and side effects of Parkinson's patients being treated with levodopa, indicating that simply increasing the capacity for dopamine synthesis in neurotypical humans is not sufficient to cause significant illusory perception in the visual modality. The authors acknowledge that the low trial number in the Snowy Pictures Task and the timing of their drug administration may limit the conclusions that they draw. However, the manuscript lacks clarity in various areas which limits the interpretability of their results.

Major comments

1. The introduction says "midbrain (striatal) dopaminergic neurons". Midbrain dopaminergic neurons are not striatal, they project to striatum. Do the authors mean to refer to the terminals of these neurons, or to the dopamine release in striatum? Apart from the Schmack et al 2021 paper, the additions to the introduction relating to learning and prediction error appear irrelevant because they are not integrated into the text. I would suggest that the authors review the literature thoroughly and use it to explain why they have designed their experiment in this way.

2. The hierarchical Bayesian modelling is not well explained in the results or methods sections, and the authors might consider including the limitations of their use of such a method as well.

3. In the limitations section there is text saying 'the timing and therefore the dose during task performance' - I am not clear what this means. Are the authors trying to say that the elimination of the drug may have resulted in their negative findings? If so, this suggests poor experimental design and makes these results very difficult to interpret.

Minor comments

1. The methods are still not completely clear to me. The authors might consider using the same task descriptions throughout the text e.g. is the 'visual perception task' the same as the 'visual discrimination task' the same as the 'snowy pictures task'? Also is the 75 minutes after intake of tablet referring to when the visual task was started or when the other tasks were started?

2. There is reference to a study on methylphenidate added to the limitations section which seemingly does not relate to the text around it.

3. There are typos throughout the text.

## Author Response

Synthesis of Reviews:

Synthesis Statement for Author (Required):

As you can see from their comments below, the reviewers appreciate the efforts that have gone into the revision of this manuscript. However, as evident from our discussion on this, we all agree the low trial number seriously limits the conclusions that can be drawn based on the presented results. As such, if further data collection to address this issue is not an option, the article needs to state clearly and early on (i.e. already in the abstract) what the limitations of this study are, and that the results need to be interpreted with caution.

Besides this, further information on the bayesian analyses conducted and some more detail in other areas is required for the article to be acceptable for publication. Please see detailed comments of the reviewers below.

Best wishes, Frederike Beyer > We now point out the limitation of the small number of trials in the Abstract: "Since the task comprised a rather low number of items, power may have been limited so as to detect small to medium effects. The results should therefore be interpreted with caution. Future studies should address illusory pattern perception using more items, and possible dose-dependent effects and differential effects in healthy vs. clinical samples." (p. 1, Abstract) > Further, we describe the hierarchical Bayesian analysis of the task in more detail (see response to reviewer #2 and Supplement).

Reviewer 1 comments Although the trial counts for each participant remain quite low, the newly added hierarchical Bayesian modelling does add more credence to the null-findings reported in this manuscript. Besides this addition, the manuscript has been sufficiently revised, particularly regarding previous literature and detailing of the methods.

Reviewer 2 comments The authors argue that levodopa has no effect on the illusory visual pattern perception in the Snowy Pictures Task. This is of potential interest to research into perception, psychosis, and side effects of Parkinson's patients being treated with levodopa, indicating that simply increasing the capacity for dopamine synthesis in neurotypical humans is not sufficient to cause significant illusory perception in the visual modality. The authors acknowledge that the low trial number in the Snowy Pictures Task and the timing of their drug administration may limit the conclusions that they draw. However, the manuscript lacks clarity in various areas which limits the interpretability of their results.

Major comments 1. The introduction says "midbrain (striatal) dopaminergic neurons". Midbrain dopaminergic neurons are not striatal, they project to striatum. Do the authors mean to refer to the terminals of these neurons, or to the dopamine release in striatum? Apart from the Schmack et al 2021 paper, the additions to the introduction relating to learning and prediction error appear irrelevant because they are not integrated into the text. I would suggest that the authors review the literature thoroughly and use it to explain why they have designed their experiment in this way. > We thank the reviewer for pointing out the imprecise wording. We edited the respective sentence accordingly: "A large body of research suggests that dopamine modulates striatal coding of prediction errors to enable learning (Basanisi et al., 2023; Glimcher, 2011; Pessiglione et al, 2006; Schlagenauf, 2013; Schultz, 2016)." > Following the reviewer's suggestion, we revised the Introduction and provide a more coherent review of the literature and reasoning for our study design (see section 1, Introduction).

2. The hierarchical Bayesian modelling is not well explained in the results or methods sections, and the authors might consider including the limitations of their use of such a method as well. > Following the reviewer's suggestion, we now describe the hierarchical Bayesian model of the SDT parameters in greater detail (see also Supplement). To summarise the edits, we provided further information on the model structure (hierarchical linear regression model), priors, and denote equations for the fixed and random effect structure. > Further, we address limitations of using hierarchical Bayesian models (see also Supplement): "Hierarchical Bayesian models also come with some limitations. Such models are sensitive to the choice of the prior distribution. Using hierarchical Bayesian modelling, we assume a specific type of distribution for the variability across the group, which may fall short in some cases (McGlothlin &Viele, 2018)." 3. In the limitations section there is text saying 'the timing and therefore the dose during task performance' - I am not clear what this means. Are the authors trying to say that the elimination of the drug may have resulted in their negative findings? If so, this suggests poor experimental design and makes these results very difficult to interpret.

Since the study was part of a larger project, the participants completed three tasks in succession. L-DOPA has a plasma half-life of approximately 90 minutes (Hauser, 2009). The pattern perception task was completed approximately 75 minutes after the intake and was typically completed in 5 to 10 minutes. Therefore, there likely was a decrease in plasma levels compared to the previous tasks, but we were still well in the time window of the plasma half-life. We now discuss this in greater detail in the limitations section: "The current study has some limitations. Plasma levels peak 30 to 60 minutes after intake of L-DOPA, while plasma half-life is around 90 minutes (Hauser, 2009; Keller et al., 2011). Being part of a larger project, the present task was performed following two other behavioural tasks. The task was completed approximately 75 minutes after intake and was typically completed in 5 to 10 minutes. Accordingly, the task was completed after plasma levels had peaked, but likely before the plasma half-life was reached. Still, the timing, and therefore the dose during task performance, may have contributed to the null effects of L-DOPA on pattern perception" Minor comments 1. The methods are still not completely clear to me. The authors might consider using the same task descriptions throughout the text e.g. is the 'visual perception task' the same as the 'visual discrimination task' the same as the 'snowy pictures task'? Also is the 75 minutes after intake of tablet referring to when the visual task was started or when the other tasks were started? > We thank the reviewer for pointing out that we need to clarify the procedure and describe this more clearly: "30 minutes prior to testing, the participants received a non-distinguishable tablet containing either 150 mg L-DOPA, a dopamine precursor, and benseracide, a peripheral decarboxylase inhibitor, or placebo, and then completed an intertemporal choice task and a reinforcement learning task [...] Approximately 75 minutes after intake of the tablet, a subset of participants additionally completed a visual perception task [...]" (section 2.2 of Methods) Also, we now consistently describe the Snowy Pictures Task as "visual perception task", and specifically refer to the task as "Snowy Pictures Task" after having described the task in detail in section 2.3.

2. There is reference to a study on methylphenidate added to the limitations section which seemingly does not relate to the text around it. > We thank the reviewer for the hint. The sentence was formulated in a misleading way. We report that in another study, the effect of enhancing dopamine transmission was found to be dose-dependent. We rephrased the sentence accordingly: "Dose-dependent effects on perceptual decision making have been reported in another study, which modulated dopamine transmission via metylphenidate (Beste et al., 2018)." 3. There are typos throughout the text. > We thank the reviewer for the hint and checked the manuscript for spelling errors.
